# A Detailed Clinical Approach to Non-dystrophic Myotonia: A Case Report of Two Brothers With Myotonia Congenita

**DOI:** 10.7759/cureus.40869

**Published:** 2023-06-23

**Authors:** Zainab Gilitwala, Shalmali Satpute, Sumant Patil

**Affiliations:** 1 Pediatrics, Rajarshi Chhatrapati Shahu Maharaj (RCSM) Government Medical College, Kolhapur, IND; 2 Pediatrics, Deenanath Mangeshkar Hospital and Research Center, Pune, IND; 3 Pediatric Intensive Care Unit, Deenanath Mangeshkar Hospital, Pune, IND

**Keywords:** non-dystrophic, mexiletine, channelopathies, herculean body, myotonia

## Abstract

Non-dystrophic myotonia (NDM) is a group of rare mono-genetic muscle disorders caused by skeletal muscle sodium or chloride channelopathies. These disorders are characterized by high muscle tone and the inability of the muscles to relax spontaneously after voluntary contraction. Myotonia congenita refers to a form of NDM that typically manifests during the later stages of childhood. It occurs as a result of genetic mutations affecting the chloride channels found in the sarcolemma membrane of skeletal muscles. Here, we present a case series of two male siblings born out of third-degree consanguineous union ages 10 and eight years, respectively, who presented with proximal muscle weakness and the characteristic “Herculean body” appearance. They demonstrated characteristic clinical diagnostic signs of myotonia. The diagnosis of myotonia congenita was confirmed through distinctive electromyography (EMG) findings, which were further supported by genetic testing revealing a homozygous mutation c.1445G>A in exon 13 of the CLCN1 gene, indicating autosomal recessive inheritance. This uncommon condition exhibits characteristic clinical manifestations and classical EMG findings, which are difficult to disregard once encountered. Genetic tests serve as a valuable tool to validate the diagnosis.

## Introduction

Myotonia congenita is a rare type of non-dystrophic myotonia (NDM). It is highly uncommon as it affects only one out of 1,00,000 children globally. It is caused by a mutation of the CLCN-1 gene on chromosome 7 that codes for voltage-gated chloride channels of the sarcolemma membrane [1]. The defective channel causes inappropriate hyperexcitability, resulting in the muscle's inability to relax following a voluntary contraction. Prolonged contraction can lead to stiffness, cramping, and muscle rigidity. In many cases, patients have abnormal enlargement of muscles which may lead to a “Herculean” or “Body-Builder” appearance. It is classified into two types - Beckers and Thomsen disease [2]. Becker is an autosomal recessive condition that is characterized by severe myotonia and permanent weakness while Thomsen disease is an autosomal dominant disease that presents with milder myotonia and mild temporary weakness [3]. Myotonia, the inability to relax, is the hallmark feature of the condition. Myotonia congenita commonly presents as difficulty walking, feeding and clumsy gait, and the inability to release hand grip. Percussion myotonia and positive Gower sign are often noticed. This condition is not fatal and muscle rigidity is the only associated complication. This case report highlights the clinical presentation of myotonia congenita and the classical electromyographic (EMG) findings which clinch the diagnosis of this extremely rare condition.

## Case presentation

Two siblings, ages 10 and eight years, born out of a third-degree consanguineous marriage, presented to the hospital with primary complaints of symmetrical proximal muscle weakness. The weakness was more pronounced in the lower limbs compared to the upper limbs, and the older sibling was more affected than the younger one. The weakness started during early childhood and has been progressively worse since then. They experience difficulty initiating and completing movements like standing from a sitting position, releasing hand grip, and have severe exercise fatigue. The parents have also observed that both of their children have well-defined bodies with rigid muscles, resembling the physique of bodybuilders. This characteristic is particularly prominent in the older brother. There is no record of any fluctuations in weakness throughout the day. The symptoms are not accompanied by pain or loss of sensation. Additionally, there is no history of fever, joint pain, rash, or bowel or bladder dysfunctions. Furthermore, there are no reports of similar complaints among other male family members. There was a history of a gross motor delay in both siblings as they started walking independently at 2.5 to three years. Currently, they have attained all milestones and there is no intellectual impairment. Upon examination, the boys were vitally stable. The brothers had a “Herculean/Body Builder” appearance. Echocardiography and thyroid studies were conducted, and both were normal. There was increased muscle tone and hypertrophy noted in their upper and lower limbs, and mild limb lordosis was also present. These findings were more pronounced in the older sibling. When assuming a sitting to standing position, the older sibling climbed to a standing position by progressively “walking” their hands up their shins, knees, and thighs, which is a positive Gowers sign. Musculoskeletal system examination in the siblings showed that muscle stiffness was reduced on repetitive movement/ exercise (warm-up phenomenon). Percussion myotonia which is a myotonic contraction in a muscle as a reaction to percussion was elicited in the calf muscles and thenar muscles. Thumb flexion was seen on tapping of the thenar eminence. Hand grip myotonia was noticed. These findings were better elicited in the older sibling. This suggested that the younger sibling had a milder phenotypic presentation (Table 1). Other systemic examinations were within normal limits. The serum creatine phosphokinase levels of both siblings were normal.

**Table 1:** Clinical features of the patients

Characteristics	Sibling 1	Sibling 2
Sex	Male	Male
Age onset	4 years of age	5 years of age
Consanguinity	Third degree	Third degree
Inheritance	Autosomal Recessive	Autosomal Recessive
Clinical Myotonia		
Tongue	Absent	Absent
Jaw muscles	Absent	Absent
Neck Muscles	Absent	Absent
Hands	Present	Present
Legs	Present	Present
Warm-up	Present	Present
Grip Myotonia	Present	Present
Triggers for Myotonia		
Cold	Present	Present
Stress	Present	Present
Fatigue	Present	Present
Exercise	Present	Present
Others		
Muscle hypertrophy	Present	Present
Muscle Pain	Absent	Absent
Transient weakness	Present	Present
Permanent weakness	Absent	Absent
Gower Sign	Present	Absent
Serum Creatine Phosphokinase Level	Normal	Normal
Diagnostic Tests		
Electromyography	Myotonic discharges ‘Dive bomber appearance’	Myotonic discharges ‘Dive bomber appearance’
Mutation in CLCN1	c.1445G>A	NA
Diagnosis	Becker Disease	Becker disease like phenotype

For both cases, the diagnosis was established with EMG which showed abnormal spontaneous repetitive muscle fiber discharge with waxing and waning frequency and amplitude (Dive Bomber’s Sound) (Video 1). Genetic testing in the older sibling reported homozygous mutation c.1445G>A affecting exon 13 of the CLCN1+ which encodes the chloride channels responsible for the myotonia congenita. The genetic testing of the younger brother was not done. This rare neuromuscular disorder does not have a definitive cure, but the use of non-pharmacological therapies can be highly advantageous in these cases. Physiotherapy was initiated for the two siblings enhancing the functioning of their limbs and reducing stiffness. Additionally, the parents were advised to exercise caution while using general anesthesia for their children, and that extra care should be taken while using depolarizing muscle relaxants during anesthesia due to the risk of life-threatening muscle spasms and secondary ventilation difficulties that can occur with the use of suxamethonium, which should be avoided in patients with myotonia congenita [3]. Other agents such as beta-agonist drugs like adrenaline and propranolol, which have been found to worsen myotonia, should also be avoided. Currently, mexiletine is the only U.S. Food and Drug Administration (FDA)-approved drug for this condition [4]. The patients were monitored monthly at the neurology clinic, and physiotherapy significantly alleviated myotonic symptoms. Due to its hereditary nature, parents were provided with genetic counseling and the option of prenatal diagnosis for future pregnancies.

**Video 1:** Dive Bomber's sound: classical EMG finding in myotonia congenita

## Discussion

NDM refers to a collection of uncommon muscle disorders resulting from sodium or chloride channelopathies in the skeletal muscles [5]. NDMs are skeletal muscle ion channel disorders that have been historically recognized as separate from myotonic dystrophy due to the lack of progressive muscle weakness and systemic symptoms [6]. NDM is characterized by myotonia, which is elevated muscle tone and the inability of muscles to naturally relax following voluntary contractions. They can be distinguished on the basis of their clinical features as shown in Table 2.

**Table 2:** Difference between dystrophic and non-dystrophic myotonias

Dystrophic Myotonia	Non-dystrophic Myotonia
Age of onset young adult	Age of onset childhood
Muscular atrophy	Muscular hypertrophy
No stiffness	Stiffness
Distal muscular weakness> Proximal muscular weakness	Proximal muscular weakness of leg
Muscular degeneration present	Non-muscular degeneration
Cardiac problems	Rarely involved
Intellectual disability common	No intellectual disability

**Figure 1:**
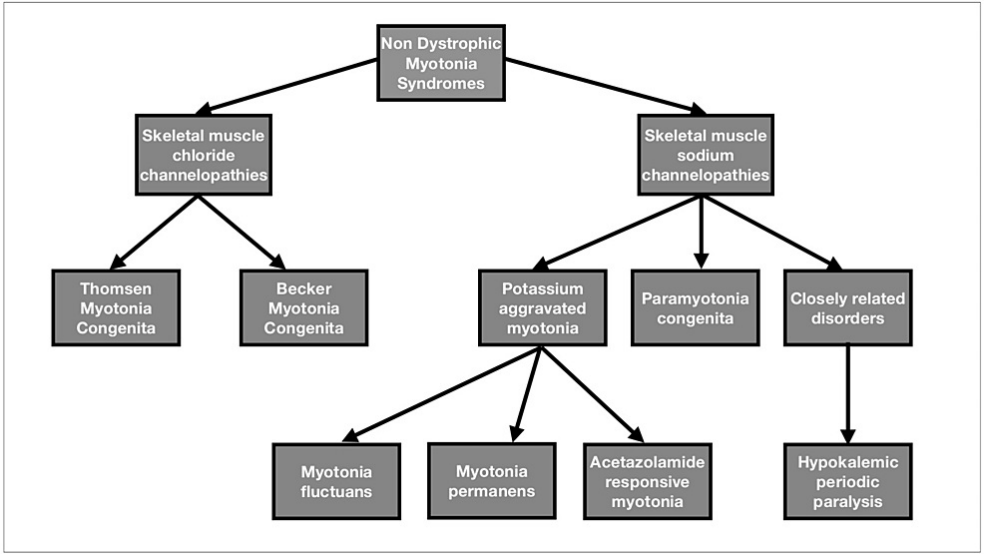
Classification of non-dystrophic myotonia syndromes

NDM is classified into two types of skeletal muscle channelopathies - chloride and sodium (Figure 1)*. *Sodium channelopathies include paramyotonia congenita (PMC) and potassium-aggravated myotonia. PMC (Eulenburg's disease) is an autosomal dominant inherited disease whose predominant feature is an episodic cold or exercise-induced muscle myotonia in exposed areas (mainly the face, neck, and hands) that lasts for minutes to hours [7]. The potassium-aggravated myotonia (PAMs) include three diseases with similar phenotypes: myotonia fluctuations, myotonia permanens, and acetazolamide-sensitive myotonia [7]. Chloride channelopathies also known as myotonia congenita are further divided into Beckers and Thomsen (Table 3). Beckers (autosomal recessive) is more aggressive causing severe symptoms and permanent weakness as compared to Thomsen (autosomal dominant) [8].

**Table 3:** Difference between sodium and chloride channelopathies

Sodium Channelopathies	Chloride Channelopathies
Lower age of onset (5 years)	Higher age of onset (10 years)
Worsening of stiffness after repetitive use (paramyotonia)	Decrease of myotonia after repetitive use (warm up phenomenon)
Presence of pain	Lack of pain
Presence of face stiffness	Presence of leg stiffness
Eyelid Myotonia	Action/Percussion myotonia in leg muscles
Long duration high amplitude myotonic potentials with a ‘slowly decelerating sound’	Short duration, low amplitude myotonic potentials with a ‘dive bomber sound’
Inter-discharge interval of myotonic potentials <30ms	Inter discharge interval of myotonic potentials >30ms

Myotonia congenita, a specific form of NDM, typically manifests in late childhood and is caused by mutations in the chloride channels found in the sarcolemma membrane of skeletal muscles [9]. Myotonia congenita is a rare cause of muscle weakness, fatigue, and pain. Multiple differential diagnoses can be considered in patients with muscle weakness including muscular dystrophies, mitochondrial myopathies, metabolic myopathies, and neuromuscular causes. Clinical findings in myotonia and specific bedside confirmatory tests are the hallmark of diagnosis. Action myotonia, which is commonly tested in handgrip and eyelid muscles, can be observed by watching for delayed muscle relaxation following 5-10 s of maximal muscle contraction [10]. It is also valuable to evaluate the warm-up phenomenon versus paradoxical myotonia, which may be a clue as to what type of channelopathy present. This is done by asking the patient to repeatedly tightly close and open their eyes or handgrip up to five times to determine whether the speed of relaxation improves (warm-up) or worsens (para myotonia) with repetition [10]. Other helpful signs include percussion myotonia in the thenar, forearm extensors, trapezius, quadriceps, and tongue muscles and lid-lag signs.

**Figure 2:**
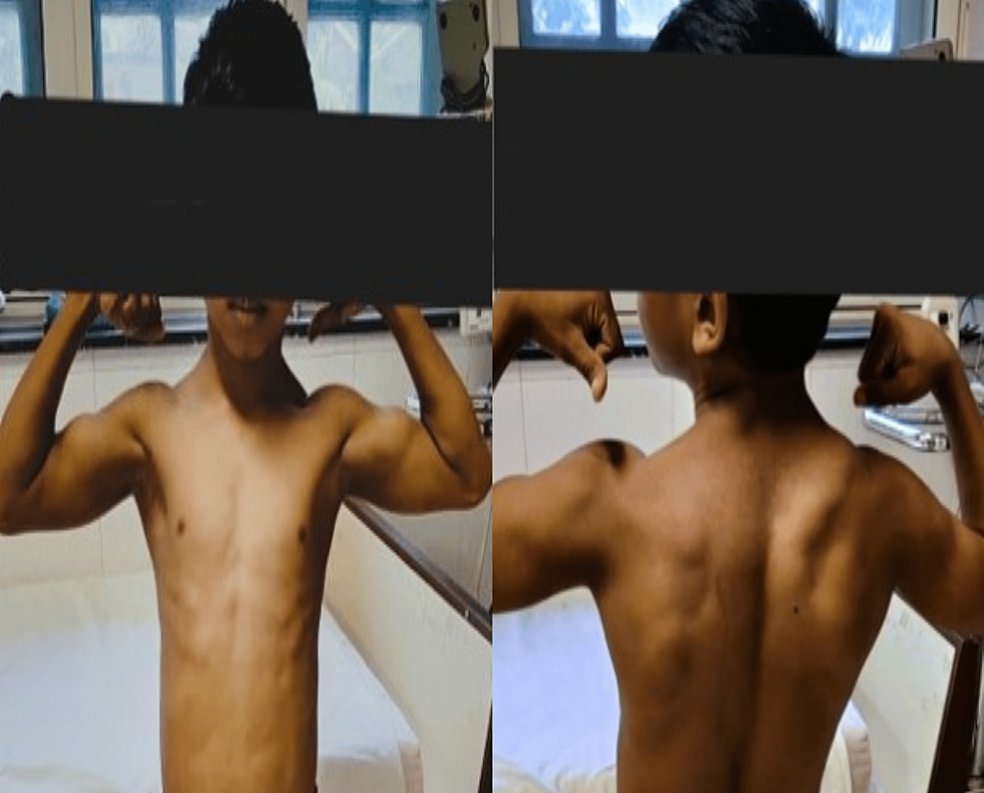
Body-builder appearance in the older sibling

EMG is a useful diagnostic tool to confirm myotonia, especially in cases where clinical symptoms are not evident. Additionally, genetic testing should be conducted to confirm the diagnosis. Since there is an overlap in the phenotypes of both sodium channelopathies (SCN4A gene) and chloride channelopathies (CLCN1 gene), testing for both genetic causes is recommended in most cases. Limited data are available regarding the detection rates of CLCN1 and SCN4A variants in patients with a strong clinical suspicion of NDM [4]. In cases where a genetic test yields negative results, it is advisable to explore alternative diagnoses. However, if the patient exhibits electrical myotonia, it is important to consider dystrophic myotonias as a possibility. Although muscle ultrasound and muscle MRI are not currently part of the diagnostic algorithm, research has been conducted to explore their potential utility. It is worth noting that a muscle biopsy is not required for diagnosing myotonia congenita [11]. Continual research is being conducted to explore treatment options for myotonia. Presently, sodium channel blockers such as Mexiletine, classified as a Class 1B antiarrhythmic, are utilized to enhance the fast inactivation of sodium channels and are regarded as the most effective form of treatment [3]. Mexiletine currently stands as the only medication approved by the FDA for addressing this specific condition [4]. Ranolazine, another sodium channel blocker typically used for chronic angina, is also used as a treatment modality. Additionally, other medications such as carbonic anhydrase inhibitors, antidepressants, and calcium channel blockers have been explored for treating myotonia [12]. In a small open-label study, patients diagnosed with myotonia congenita demonstrated improvement in myotonia symptoms when administered acetazolamide, a carbonic anhydrase inhibitor. For pediatric cases, carbonic anhydrase is often the preferred initial treatment due to its safety profile [10]. In addition to medical intervention, genetic counseling, and physiotherapy are recommended. Surprisingly, despite all patients reporting symptoms, 39% of individuals are not receiving any anti-myotonic treatment [5]. The reasons behind this discrepancy are not entirely clear. It could be attributed to milder symptoms that did not necessitate treatment, limited awareness among physicians regarding available treatment options, reluctance from both physicians and patients to initiate “cardiac drugs” like mexiletine, or issues related to medication efficacy, side effects, or insurance coverage (Figure 2) [4].

## Conclusions

Myotonia congenita is a rare type of neuromuscular channelopathy characterized by unique clinical features and distinctive signs of myotonia. It is associated with a bodybuilder appearance and characteristic signs that can be observed through electromyography (EMG), which firmly establishes the diagnosis. The presence of a specific mutation is confirmed through genetic testing. However, due to the disease's rarity, research and clinical trials exploring treatment options remain limited. Presently, mexiletine stands as the sole FDA-approved medication available. Unfortunately, a treatment gap persists in addressing these conditions, leaving many patients untreated. Further investigations are needed to bridge these gaps and enhance patient satisfaction with treatment. Currently, patients receive regular physiotherapy and genetic counseling for both parents and family members.
